# Cytotoxic innate lymphoid cells sense tumor-derived IL-15: a novel mechanism of cancer immunosurveillance

**DOI:** 10.1038/s41392-022-01173-x

**Published:** 2022-09-17

**Authors:** Tobias Franz, Jonas Negele, Sascha Kahlfuss

**Affiliations:** 1 Institute of Molecular and Clinical Immunology, Medical Faculty, Otto-von-Guericke University Magdeburg, Magdeburg, Germany; 2 Institute of Medical Microbiology and Hospital Hygiene, Medical Faculty, Otto-von-Guericke University Magdeburg, Magdeburg, Germany; 3 Health Campus Immunology, Infectiology and Inflammation (GCI3), Medical Faculty, Otto-Von-Guericke University Magdeburg, Magdeburg, Germany; 4 Center for Health and Medical Prevention (CHaMP), Otto-von-Guericke-University, Magdeburg, Germany

**Keywords:** Translational immunology, Tumour immunology

Recently, Li and colleagues provided in *Nature immunology*^1^ a novel mechanism of immunosurveillance in epithelial tumors, in which cytotoxic innate lymphoid cells (ILCs) sense IL-15 secreted by the tumor itself.

Both the adaptive immune system, including CD8^+^ cytotoxic lymphocytes (CTLs), and innate immune cells such as Natural Killer (NK) cells are capable of providing antitumor immunity through the release of perforins and granzymes. In addition, also cytotoxic group 1 innate lymphoid cells (ILC1s) were reported to exert NK cell-like antitumor cell cytotoxicity.^2^

Kansler et al. now reveal that tumor-derived IL-15 is an important prerequisite for the cytotoxic function of ILC1s during renal cell carcinoma (RCC), which histologically can be subdivided into chromophobe RCC (chRCC) and clear cell RCC (ccRCC). By performing single-cell RNA sequencing (scRNAseq) of all CD45^+^ cells from ccRCC and chRCC the authors first identified distinct CD8^+^ T cell clusters between both RCC subtypes. Validation experiments using flow cytometry showed that ccRCC tumors are especially characterized by an infiltration of an exhausted PD1^+^CD8^+^ T cell population, whereas this population was not significantly increased in chRCC. Further, the authors detected circulating NK cells and tissue-resident ILC1s in both types of RCC. However, the frequencies of these innate lymphocyte populations were increased only in chRCC but not in ccRCC. In addition, ILC1s in chRCC, but not in ccRCC, expressed higher levels of CD56, a marker enriched in tissue-resident ILCs. Together, these findings indicate that the immune cell landscape and, consecutively, the antitumor response in chRCC compared to ccRCC appear to be different.

Importantly, using The Cancer Genome Atlas (TCGA) the authors showed that an immune cell composition in the tumor dominated by PD1^+^CD8^+^ T cells is negatively correlated with patient survival in both ccRCC and chRCC. However, ILC1 gene signatures correlated with either better (chRCC) or worse (ccRCC) outcomes. Further, ILC1s in chRCC, in contrast to ccRCC, selectively expressed high levels of granzyme A. In addition, NK cells and ILC1s showed an increased expression of the IL-2/IL-15 receptor β chain and chRCC tumors from TCGA cohort exhibited increased expression of IL-15, which is capable of enhancing anti-tumor responses.^1^

To prove whether IL-15 directly affects ILC1s, the authors cultured ILC1s from RCC tumors in the presence of IL-15/IL-15Rα complexes: Here, IL-15/IL-15Rα complexes increased granzyme A and CD56 expression, proliferation, as well as cytotoxicity of ILC1s, indicating that IL-15 is a crucial regulator of ILC1-mediated cancer immunosurveillance. Next, Kansler et al. sought to test whether these mechanisms also occur in other epithelial carcinomas. Using data from TCGA, they discovered that also in breast cancer driven by mutations in PIK3CA the ILC1 signature was positively associated with IL-15 levels and a better survival.

Finally, the authors wanted to uncover the primary source of IL-15 within the tumor micromilieu. To this end, they used different reporter mouse strains and found that IL-15 is produced by several immune cells, stromal cells, but also endothelial cells in murine polyomavirus middle tumor antigen (PyMT) tumors. However, IL-15 production by dendritic cells, macrophages, hematopoietic cells, and stromal cells appeared to be dispensable for the infiltration and function of ILC1s in PyMT tumors. Instead, the authors revealed that granzyme C expression is unique to ILC1s and that granzyme C-expressing ILC1s in PyMT tumors (and ILC1s in chRCC) closely interact with E-cadherin-expressing cancer cells. Here, ILC1s show Ca^2+^ influx, indicating their activity within the tumor microenvironment. Furthermore, IL-15 production by CD24^+^CD29^+^EpCAM^+^ epithelial cells was higher in PyMT tumors than in normal tissue and the deletion of tumor-expressed IL-15 resulted in a decrease of ILC1s, a downregulation of granzyme B and C expression, and a reduced tumor control.

**Fig. 1:**
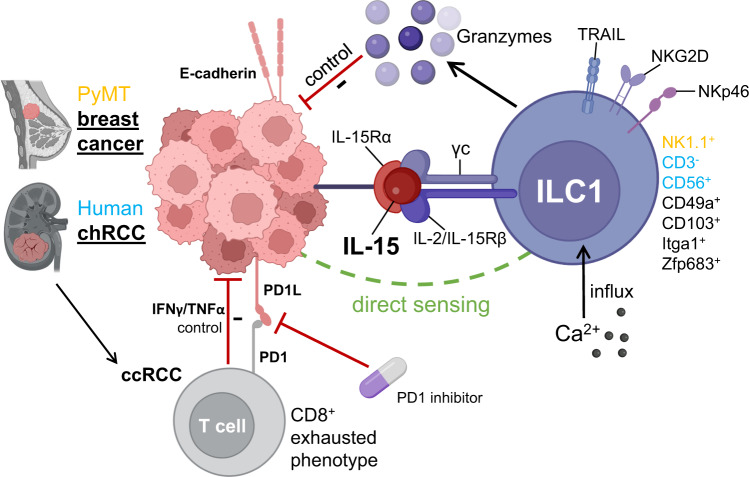
Mechanism of ILC1 tumor immunosurveillance in renal cell carcinoma (RCC) and polyomavirus middle tumor antigen (PyMT) breast cancer: Human clear cell renal cell carcinoma (ccRCC) tumors are infiltrated by exhausted PD1+CD8+ T cells, making immune checkpoint blockade an effective therapy for ccRCC patients. Instead, human (purple) chromophobe cell RCC (chRCC) and murine (yellow) polyomavirus middle tumor antigen (PyMT) tumors are characterized by infiltration of ILC1s. E-cadherin expressing cancer cells produce IL-15 and regulate the expansion of tissue-resident cytotoxic ILC1s via IL-2/IL-15 receptor β chain activation, which secrete granzymes and hence control chRCC and PyMT tumor growth. The interaction of ILC1s with tumor cells is characterized by direct sensing, Ca2+ influx, and also the expression of TRAIL, NGK2D and NKp46 on ILC1s. The figure was in part created with Biorender

Together, the current study by Li and colleagues establishes IL-15-sensing tissue-resident cytotoxic ILC1s as a conserved mechanism of epithelial cancer immunosurveillance in mice and humans (Fig. 1). In this context it needs to be emphasized that ILC1s also have the potential to promote tumor growth depending on the tissue context and ILC1 phenotype, e.g. as it was shown for fibrosarcoma progression in a murine cancer model.^1,3^ Here, a mechanism of tumor immunoevasion was demonstrated in which TGFβ-driven conversion of NK cells into ILC1s leads to a suppressed antitumor immune response and thus enhanced tumorigenesis.^4^ Future studies are needed to unravel the heterogeneous functional programs and plasticity of tissue-resident ILC1s within different types of cancer. After ILC1s have arisen in the periphery, it is plausible that they acquire a spectrum of organ- and cancer-specific effector phenotypes through a unified Hobit-dependent differentiation pathway that is fine-shaped by local factors, e.g., TGFβ or, as shown by the authors, cancer-derived IL15.^4^ Besides the IL-2/IL-15R β chain also other surface molecules, such as TRAIL, NKG2D, and NKp46 are involved in the sensing and elimination of malignant cells. Importantly, all these molecules may serve as diagnostic markers for the tumoricidal potential of innate lymphoid cells in the tumor micromilieu.

From a clinical perspective, a better understanding of innate lymphocytes and tumor-derived cytokines, such as IL-15, is key to establish more effective cancer immunotherapies. Meanwhile, several preclinical studies have addressed the use of heterodimeric IL-15 in cancer immunotherapy. However, so far the short in vivo half-life and potential toxicity associated with high doses of IL-15 still display challenges, which need to be overcome in the next years.^5^
